# Nurses in China lack knowledge of inhaler devices: A cross-sectional study

**DOI:** 10.3389/fphar.2023.1152069

**Published:** 2023-04-07

**Authors:** Ning Xie, Zheng Zheng, Qilian Yang, Man Li, Xiaofen Ye

**Affiliations:** ^1^ Department of Pharmacy, Qingpu Branch of Zhongshan Hospital, Fudan University, Shanghai, China; ^2^ Department of Nursing, Zhongshan Hospital, Fudan University, Shanghai, China; ^3^ Department of Pharmacy, Minhang Hospital, Fudan University, Shanghai, China; ^4^ Department of Pharmacy, Zhongshan Hospital, Fudan University, Shanghai, China

**Keywords:** inhaler devices, chronic disease, community hospital, nurses, knowledge of inhalation therapy

## Abstract

**Objective:** To understand the level of knowledge about inhaler devices among medical staff.

**Methods:** This study evaluated the knowledge of inhalation therapy and the use of inhaler devices among nurses in China. We administered a new self-designed online questionnaire to 1,831 nurses. The questionnaire comprised 11 questions, including the storage location of inhaler devices, steps involved in using inhaler devices, and common errors when using various devices.

**Results:** Among the 1,831 participants, 816(44.57%), 122(6.66%), and 893(48.77%) nurses worked in community, secondary, and tertiary hospitals, respectively. Adequate knowledge of inhaler devices was demonstrated by 20.10%, 8.20%, and 13.10% of nurses working in community, secondary, and tertiary hospitals, respectively. Of the nurses working in community hospitals, 27.70% knew the key points for using inhalers compared to 15.57% in secondary hospitals and 23.18% in tertiary hospitals (*p* < 0.01). Only 9.50%–26.00% of participants chose correct answers to the 9 questions about the use of inhalers. The accuracy rate of the responses was generally low, and the highest accuracy rate was 26.00%.

**Conclusion:** Knowledge of inhalation therapy was better among nurses working in community hospitals than among those working in high-level hospitals. This is because of the clearer division of work and higher workload in high-level hospitals. Overall, nurses’ knowledge of inhalation therapy is low. Furthermore, knowledge about inhaler devices should be strengthened among nurses in Chinese hospitals. It is necessary to create training opportunities for nurses in China to increase their awareness and knowledge regarding the management of chronic respiratory diseases.

## Introduction

Non-communicable diseases are the leading cause of death worldwide. Chronic respiratory diseases are among the leading causes of morbidity and mortality, and their prevalence is expected to increase in the coming years (World Health Organization, 2007; GBD Chronic Respiratory Disease Collaborators, 2020). In a national cross-sectional study, the overall prevalence of asthma in China was 4.2%, representing 45.7 million Chinese adults (Huang et al., 2019). The overall prevalence of spirometry-defined chronic obstructive pulmonary disease (COPD) in China is 8.6%, accounting for 99.9 million people with COPD (Zhu et al., 2018). In people aged ≥40 years, the prevalence was higher (13.7%) than that in the Chinese national survey conducted in 2007 (8.2%) (Wang et al., 2018).

Although they have become a major public health problem, chronic respiratory diseases are often incompletely treated. The asthma control level improved from 28.7% in 2007–2008 to 39.2% in 2015–2016 (*p* < 0.01), whereas there was no significant improvement in the rate of peak flow meter usage in the same period (Lin et al., 2018). A national survey across seven provinces found that the regular medical treatment rate of COPD patients was only 7.9% (Zhong et al., 2007). It is crucial to increase awareness of disease and self-management among patients to reduce the disease burden.

Efficient delivery of inhaled medication is essential for the success of chronic respiratory disease therapy. Thus, the correct inhaler technique is crucial as it can impact the efficient delivery of inhaled medication. A poor inhaler technique causes uncontrolled chronic respiratory disease, as failure to effectively deliver the medication to the lungs compromises its clinical efficacy (Manjulakshmi et al., 2019). Several patients with chronic airway disease do not use inhaler devices properly, which contributes to poor disease control (Anna et al., 2021). A previous study showed that non-adherent patients to treatment were almost twice as likely to die compared to adherent patients (Aramís et al., 2021).

To increase clinical efficacy, it is essential to ensure that the patient understands the correct inhaler technique. Studies have shown that patients often get confused about various attributes of different inhalers, and poor technique is common regardless of the device used (Mark et al., 2019; Sarah et al., 2021). Therefore, medical staff play a critical role in educating patients on appropriate inhaler use and ensuring medication adherence. Particularly in China, nurses are responsible for educating patients on using inhaler devices.

Therefore, we surveyed nurses’ knowledge of inhalation therapy and the use of inhaler devices in China. The results of this study provide basic data for recommendations regarding physician education in this arena.

## Materials and methods

### Selection of participants

A total of 1,831 nurses working in community, secondary, and tertiary hospitals in the Yangtze River Delta Integration Model area, China, were selected for this study. The researcher visited each clinical unit in each hospital and participated in ward meetings. After each meeting, the researcher requested all nurses to fill out an online questionnaire according to their wishes. The nurses participating in the study included personnel from all departments of the hospitals, including the department of respiratory medicine. Finally, the researcher collected the responses to the questionnaire from the web.

Questionnaire respondents were included in the study based on the following criteria: nurses who had worked in public hospitals in the Yangtze River Delta Integration Model area, China; provided informed consent; and voluntarily participated in this study. The exclusion criteria were as follows: personnel on vacation or sick leave and those not willing to participate in the research.

### Questionnaire

We designed a new questionnaire to assess the knowledge of inhaler devices. The questionnaire was verified by experts in the fields of respiratory medicine, nursing, and pharmacy. About 5 doctors, 10 pharmacists, and 10 nurses participated in the design of the questionnaire, which was checked and revised repeatedly. The questionnaire consisted of two parts. The first part recorded participants’ age, sex, education level, and contact information. The second part consisted of 11 questions on all types of inhaler devices. The first question concerned the main route of drug administration for asthma and COPD. The second question concerned the storage location of the inhaler devices. The third question asked participants to list the steps involved in using inhaled drugs. The fourth and fifth questions were about the main points in using the metered dose inhaler (MDI) and dry-powder inhaler (DPI), respectively. Questions 6–11 asked participants to identify the wrong options when using Turbuhaler, Accuhaler, Ellipta, HandiHaler, Respimat, and Easyhaler, respectively. The study was conducted online from October 2020 to January 2021.

### Statistical analysis

Statistical analyses were performed using SPSS Statistics for Windows, version 21.0 (IBM Corp., Armonk, NY, United States). Socio-demographic characteristics and other categorical variables are presented as frequencies and percentages. The chi-squared test was used to test for differences. The significance level was set at *p* < 0.05.

## Results

### Baseline characteristics

Table 1 shows the participants’ sociodemographic characteristics. A total of 751 (41.02%) nurses were aged between 20 and 30 years. Most participants were women (98.69%) and came from province-level municipalities (87.39%). The numbers of nurses working in community, secondary, and tertiary hospitals were 816 (44.57%), 122 (6.66%), and 893 (48.77%), respectively. Most nurses held junior (49.65%) and intermediate (32.22%) positions. Their education level was mainly undergraduate (54.18%) and junior college (41.23%), and most (74.71%) had never worked in the department of respiratory medicine.

**TABLE 1:** Sociodemographic characteristics of participants.

Characteristics of nurses (*n* = 1,831)	Frequency	Percent (%)
Recorded age in years		
<20 years	20	1.09
≥20 years but <30 years	751	41.02
≥30 years but <40 years	627	34.24
≥40 years	433	23.65
Sex		
Male	24	1.31
Female	1,807	98.69
Region		
Province-level municipality	1,600	87.39
Provincial capital	46	2.51
Prefecture-level city	46	2.51
County-level city or below	139	7.59
Hospital level		
Tertiary hospital	893	48.77
Secondary hospital	122	6.66
Community hospital	816	44.57
Professional title		
Titles below junior title	318	17.37
Junior title	909	49.65
Intermediate title	590	32.22
Senior title	14	0.76
Education		
Senior school or below	78	4.26
Junior college	755	41.23
Undergraduate	992	54.18
Postgraduate	6	0.33
Working years		
<5 years	445	24.30
≥5 years but <10 years	451	24.63
≥10 years but <20 years	526	28.73
≥20 years	409	22.34
Major in respiratory medicine		
Never	1,368	74.71
Once	346	18.90
Current	117	6.39

### Inhaler device storage

**FIGURE 1:**
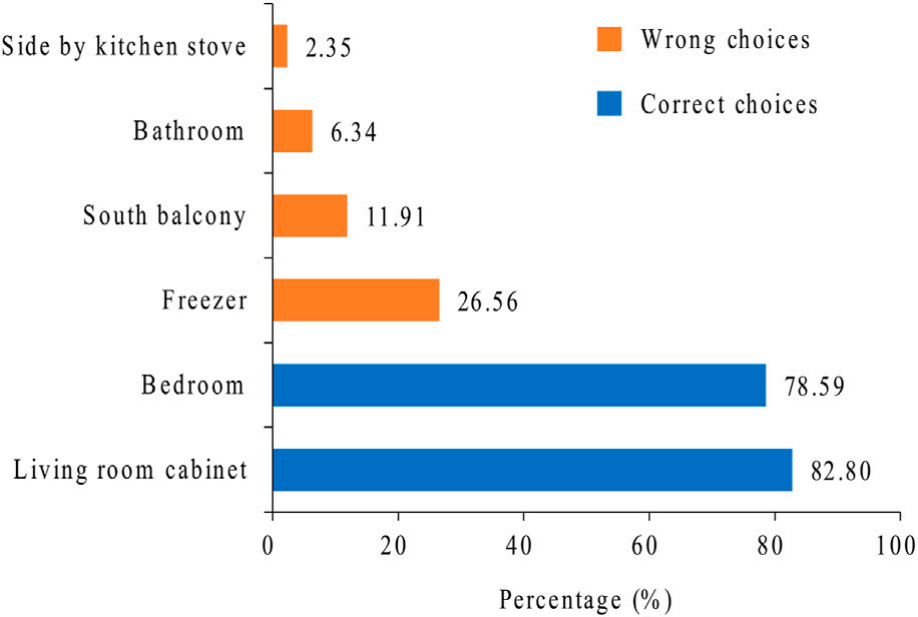
Storage place of inhaler devices after opening. The numbers indicate the percentage of the selection. The two blue strips represent the correct choices.

As shown in Figure 1, inhaler devices should be stored in a living room cabinet or the bedroom after opening. The percentage of nurses choosing two options was 82.8% and 78.59%, respectively. A total of 50.52% of the nurses chose both options, and 49.48% chose unsuitable storage locations (such as the bathrooms, balconies, kitchens, or refrigerators) for inhaler devices.

### Knowledge of inhaler devices

**FIGURE 2:**
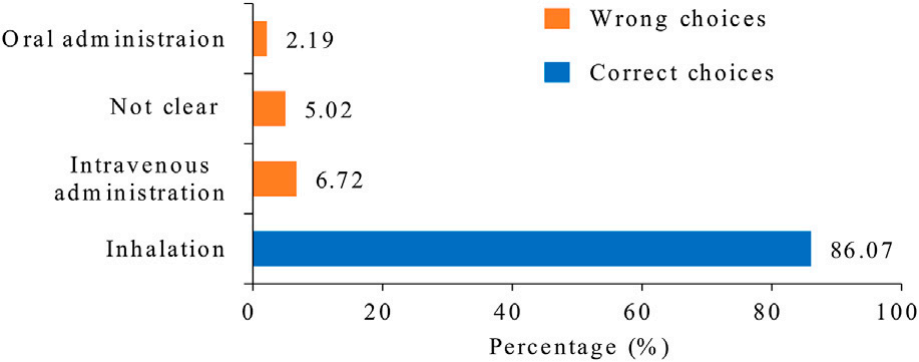
Main route of drug administration for asthma and COPD. The numbers indicate the percentage of the selection. The blue strip represents the correct choice. COPD, chronic obstructive pulmonary disease.

Figure 2 shows that 1,576 (86.07%) nurses selected inhalation as the main route of drug administration for asthma and COPD, and 13.93% incorrectly selected oral, intravenous, or other routes as the main route of administration.

**FIGURE 3:**
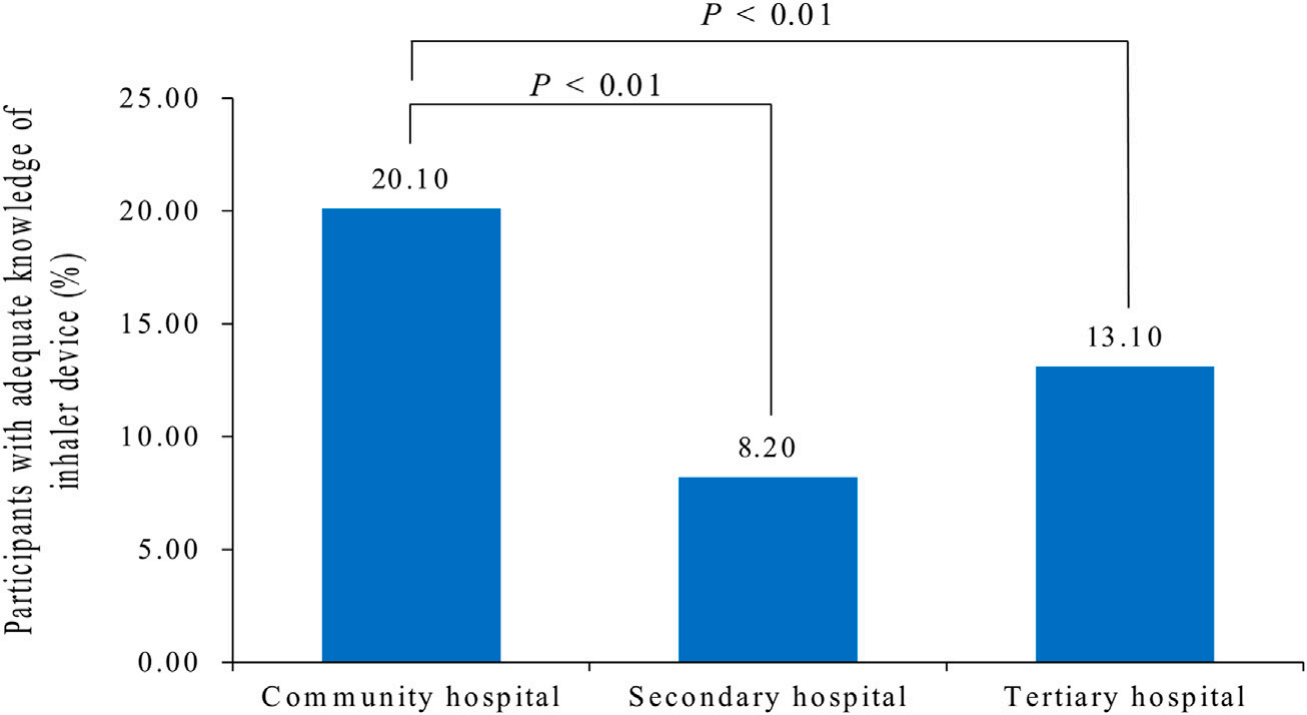
Percentage of participants with adequate knowledge of inhaler devices. There are significant differences between community and secondary/tertiary hospitals (*p* < 0.01).

Supplementary Table S1 shows the sociodemographic characteristics and knowledge of the inhaler devices. Adequate knowledge of inhaler devices was demonstrated by 20.10%, 8.2%, and 13.1% of nurses working in community, secondary, and tertiary hospitals, respectively, and the difference was statistically significant (*p* < 0.01) (Figure 3). Only 54 of 117 nurses working in the department of respiratory medicine had adequate knowledge of inhalers. The knowledge of inhaler devices was significantly different between nurses working in the department of respiratory medicine and those who had never worked in the department of respiratory medicine (*p* < 0.01).

### Technique of inhaler use

**FIGURE 4:**
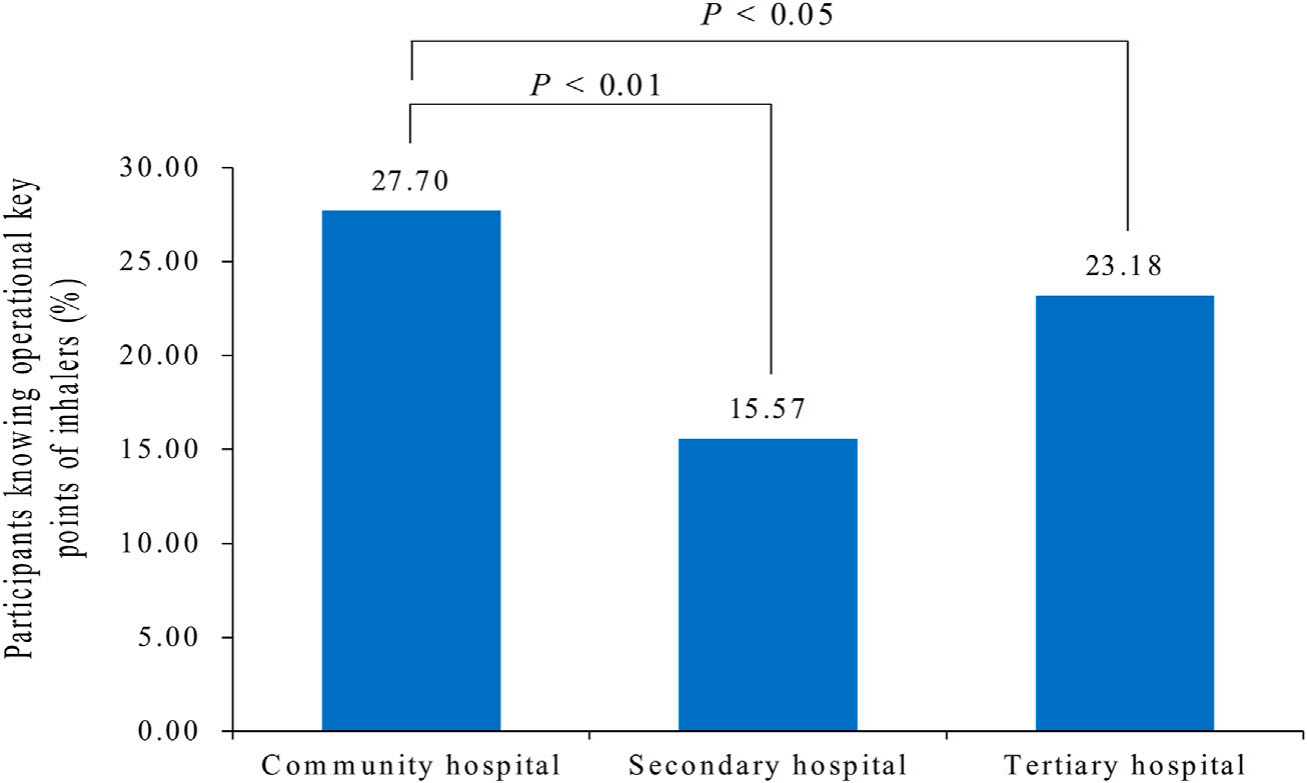
Percentage of participants knowing operational key points of inhalers (MDI and DPI). There are significant differences between community and secondary (*p* < 0.01)/tertiary hospitals (*p* < 0.05). MDI, metered dose inhaler; DPI, dry-powder inhaler.

As shown in Supplementary Table S2, a slightly higher proportion (27.70%) of nurses in community hospitals knew the operational key points of using inhalers (MDI and DPI) than those in secondary hospitals (15.57%) and tertiary hospitals (23.18%). This difference was statistically significant (Figure 4). The proportions of nurses holding senior (28.57%) and intermediate (26.44%) positions who knew the operational key points were higher than those of nurses with junior (23.32%) and below junior (25.16%) positions.

As shown in Table 2, the main points of MDI include inhalation and pressing, being synchronized, slow and deep inhalation, and 21.41% of participants chose these two points. DPI does not require hand–mouth synchronization but need inhalation hardly and deeply, and 9.50% of participants chose these two points. Only 114 (6.23%) nurses answered both questions correctly.

**TABLE 2:** Overview of the questions about knowledge of inhaler use.

Items in the questionnaire	Answer	Correct answer	Number of correct answer (%)	Number of wrong and unknown answers (%)
Q1: Please list the steps of using inhaled drugs (fill in the blank)	①Breath out. ② Remove or turn cover correctly and insert capsule. ③Deep mouthwash. ④Breath in. ⑤Hold the breath	②①④⑤③	464 (25.34%)	1,367 (74.66%)
Q2: What are the main points that need to be followed when using MDI? (multiple choice)	A. Unclear. B. Inhale and press simultaneously. C. It is not necessary inhale and press simultaneously. D. Inhale hardly and deeply. E. Inhale quick. F. Inhale slow and deep	B, F	392 (21.41%)	1,439 (78.59%)
Q3: What are the main points that need to be followed when using DPI? (multiple choice)	A. Unclear. B. Inhale and press simultaneously. C. It is not necessary inhale and press simultaneously. D. Inhale hardly and deeply. E. Inhale quickly. F. Gently inhale	C, D	174 (9.50%)	1,657 (90.50%)
Q4: Which of the following is the wrong option when using Turbuhaler? (single choice)	A. Inhaler upright during firing. B. You cannot wash the suction nozzle even though itis dirty. C. The base needs to rotate one back and forth. D. Rotate the base to reset after inhaling. E. Unclear	D	302 (16.49%)	1,529 (83.51%)
Q5: Which of the following is the wrong option when using Accuhaler? (single choice)	A. Medicine is ready to inhale after pushing the slider to the end. B. Push slider and inhale simultaneously. C. Accuhaler should be parallel to the ground when pushing the slider. D. Push the Accuhaler’s shell to close after inhaling. E. Unclear	B	424 (23.16%)	1,407 (76.84%)
Q6: Which of the following is the wrong option when using Ellipta? (Single choice)	A. Repeatedly switching the dispenser lid without medication does not cause drug wastage. B. Opening the dispenser lid indicates a suction is ready. C. Cannot breathe into the suction nozzle of the device. D. It is unnecessary to breathe in and open the suction nozzle simultaneously. E. Unclear	A	476 (26.00%)	1,355 (74.00%)
Q7: Which of the following is the correct option when using HandiHaler? (single choice)	A. For the convenience of the elderly, 10 capsules are removed from the aluminum foil. B. HandiHaler cannot be washed with water. C. Repeatedly pressing the needle is more beneficial for inhalation. D. The sound of capsules rotating can be heard while inhaling. E. Unclear	D	434 (23.70%)	1397 (76.30%)
Q8: Which of the following is the correct option when using Respimat? (single choice)	A. After the bottle is inserted into the inhaler prior to initial use, it can be removed and cleaned during use. B. Inhale and press simultaneously. C. First open the dust cover, then rotate the transparent base for half a circle until clicking. D. Hard and deep inhale. E. Unclear	B	189 (10.32%)	1,642 (89.68%)
Q9: Which of the following is the correct option when using Easyhaler? (single choice)	A. Shake the device vertically 4–5 times. B. Inhale after shaking. C. Bring the suction nozzle out of mouth after inhaling and hold breath. D. Inhale and press simultaneously. E. Unclear	A	455 (24.85%)	1,376 (75.15%)

### Knowledge of inhaler use

As shown in Table 2, there were nine questions about the knowledge of inhaler use. The first question asked participants to list the steps involved in using inhaled drugs. Only 25.34% of the nurses got this correct. The second and third questions pertained to the main points of using MDI and DPI, respectively. Question 6 (Which of the following are the wrong options when using Ellipta?) had the highest percentage of correct answers (26.00%). The question with the lowest rate of correct answers (9.50%) was question 3 (What are the main points that need to be followed when using DPI?). Regarding the use of different inhaler devices in this survey, the maximum number of correct answers was received for Ellipta (26.00%) and the minimum was for Respimat (10.32%).

## Discussion

This survey showed that a very high proportion of nurses who worked in hospitals in China lack adequate knowledge of inhaled therapy and related patient education. Overall, 463 (25.29%) nurses had past or current experience in the department of respiratory medicine. Owing to an aging population, an increasing number of elderly patients require medical services in the hospital. Thus, patients with chronic airway disease may be presented to any clinical department, not just the department of respiratory medicine. Therefore, nurses in other clinical departments also need to be trained in using inhalers.

Many patients with chronic airway disease achieve incomplete treatment benefits, due to poor disease control and adherence (Pearce and Fleming, 2018; López-Campos et al., 2019). The reasons for poor disease control and adherence include the type of medication, dosing frequency, patients’ awareness of control, and various patient beliefs and sociocultural and psychological variables (Jordi et al., 2020). Adherence is a major problem for all chronic diseases. However, the inappropriate use of inhalers poses additional difficulties in patients with asthma and COPD. Several patients do not receive appropriate inhaler training, and their inhaler techniques are not monitored. The main reason for the current situation is that physicians and nurses who prescribe or supervise these inhalers have poor knowledge and skills regarding their use (Bamidele et al., 2018; Maepa et al., 2019). Studies have shown that patients with good adherence have a 44% lower rate of severe exacerbation (Jose and Oliver, 2019).

Hospitals in China are divided into three levels according to the National Health Commission of China: community, secondary, and tertiary hospitals. The national roadmap, the Healthy China 2030 plan (CPC, 2016), highlights the important role of primary healthcare in substantially strengthening chronic disease management (Gao et al., 2021). In China, especially in the community hospital, nurses usually teach patients about inhaler devices. Our study found that 44.57% of the participants worked in community hospitals. Moreover, they had better knowledge of inhaler devices than nurses in secondary and tertiary hospitals. The number of nurses who knew at least two essential steps in inhaler use was higher in community hospitals than that in secondary and tertiary hospitals. This result is consistent with Chinese national conditions. This may be because the division of labor in secondary and tertiary hospitals in China is relatively fine, leading to a lack of knowledge among non-respiratory nurses. Nurses holding junior and below positions often undertake more clinical work, while nurses holding intermediate and senior positions undertake some management work. It is suggested that nurses from non-respiratory wards, such as the department of cardiology and geriatric medicine, be trained in the department of respiratory medicine to provide inhalation education, to ensure good education for patients with non-respiratory wards, and to gain medication safety.

Our questionnaire differed from other questionnaires where it focused on the errors that patients tend to make when using various inhalation devices. Our study included the important points of commonly used inhalers. It was more friendly and concise, and we easily obtained the cooperation of the responders. In contrast, other questionnaires are more complicated, assess only the types of inhalers, and no specific device is evaluated. Regarding the composite variable, general knowledge of inhaled therapy, we pooled the answers to the questions about the use of inhaler devices; only 9.50%–26.00% of participants in our study responded correctly to the 9 questions. Our results indicate that the nurses’ knowledge of inhalers is not sufficient for effective communication with patients. This finding is similar to the results of other studies. Hajed evaluated that the healthcare providers’ knowledge of aerosol drug delivery is completely inadequate. There is an urgent need to introduce an aerosol drug delivery educational package in the curricula (Al-Otaibi, 2020). Another study showed that the general level of knowledge regarding the use of inhalation devices is low among nurses working in this field in Spain (Jordi et al., 2016). Vinita et al. (2021) reported that even nurses in the department of respiratory medicine did not fully master all inhaler devices. This study revealed a strong correlation between the number of inhalers prescribed in the previous year and nurses’ inhaler device scores. Our previous study demonstrated that inhaler training can increase the patients’ peak inhalation flow rate and improve their ability to use DPIs (Hua et al., 2021). Thus, educating healthcare workers is vital for teaching patients about the correct use of inhaler devices.

In this study, we identified that there is a lack of knowledge about inhaler devices among nurses in China. Based on the current situation, it is necessary to create training opportunities for nurses to increase their knowledge of inhaler devices. In addition, we should establish a team of professionals to regularly measure nurses' knowledge of inhaler devices. Regular assessment and ongoing education on the correct inhaler technique for respiratory nurses are necessary to optimize device usage by nurses. Finally, related institutional improvements are important. Chronic diseases require long-term management, including patient self-management based on accurate knowledge of drug use and regular review at the hospital. The hierarchical medical system in China is a good way to facilitate the medical treatment of patients with chronic diseases in community hospitals. Many patients with chronic diseases receive medication guidance in community hospitals. As some tests are only performed in tertiary hospitals, nurses in tertiary hospitals must master the key points of medication for chronic diseases.

This survey is the first domestic exploration of the problem of medication errors in inhalation preparations in China. It is a comprehensive survey of nurses in community, secondary, and tertiary hospitals. However, this study had some limitations. First, all participants were nurses. Second, participants were not screened, and nurses not working in the department of respiratory medicine or who have not been exposed to inhaler devices may not have sufficient knowledge in this regard. Third, the questionnaire was completed once without repeated verification. It may be necessary to expand the inclusion criteria to include physicians and pharmacists in future studies, so that more accurate and relevant data are obtained.

In conclusion, nurses lack knowledge of inhaler devices. Nurses working in community hospitals had better knowledge about handling of inhaler devices than those in secondary and tertiary hospitals. Therefore, sufficient professional training focused on inhaler devices is urgently needed. Based on the current situation in China, we also proposed specific measures to solve this problem. Although the findings of our study are preliminary, they may provide a possible explanation for the current issues with inhaler use.

## Data Availability

The original contributions presented in the study are included in the article/Supplementary Material; further inquiries can be directed to the corresponding author.

## References

[B1] Al-Otaibi H. M. (2020). Evaluation of health-care providers’ knowledge in the science of aerosol drug delivery: Educational sessions are necessary. J. Fam. Community Med. 27 (1), 62–66. 10.4103/jfcm.JFCM_138_19 PMC6984029 32030080

[B2] Anna V. Paulien van der W. Barbara P. Lies L. (2021). Determinants of poor inhaler technique and poor therapy adherence in obstructive lung diseases: A cross-sectional study in community pharmacies. BMJ Open Respir. Res. 8 (1), e000823. 10.1136/bmjresp-2020-000823 PMC8351493 34362761

[B3] Aramís T. A. M. Charleston R. P. Antônio C. M. L. Lindemberg A. C. Gisélia S. S. Eduardo M. N. (2021). Evidence of the association between adherence to treatment and mortality among patients with COPD monitored at a public disease management program in Brazil. J. Bras. Pneumol. 48 (1), e20210120. 10.36416/1806-3756/e20210120 34909924 PMC8946558

[B4] Bamidele O. A. Ayodeji M. A. Olayinka S. I. Daniel O. O. Olubukola O. A. Gregory E. E. (2018). Knowledge of spacer device, peak flow meter and inhaler technique (MDIs) among health care providers: An evaluation of doctors and nurses. Ghana Med. J. 52 (1), 15–21. 10.4314/gmj.v52i1.4 30013256 PMC6026947

[B5] CPC (2016). The plan for “Healthy China 2030”. Available at: http://www.gov.cn/xinwen/2016-10/25/content_5124174.html (Accessed March 15, 2022).

[B6] Gao C. Xu J. Liu Y. Yang Y. (2021). Nutrition policy and Healthy China 2030 building. Eur. J. Clin. Nutr. 75 (2), 238–246. 10.1038/s41430-020-00765-6 33219269

[B7] GBD Chronic Respiratory Disease Collaborators (2020). Prevalence and attributable health burden of chronic respiratory diseases, 1990-2017: A systematic analysis for the global burden of disease study 2017. Lancet Respir. Med. 8 (6), 585–596. 10.1016/S2213-2600(20)30105-3 32526187 PMC7284317

[B8] Hua J. Ye X. Du C. Xie N. Zhang J. Li M. (2021). Optimizing inhalation therapy in the aspect of peak inhalation flow rate in patients with chronic obstructive pulmonary disease or asthma. BMC Pulm. Med. 21 (1), 302. 10.1186/s12890-021-01674-5 34560863 PMC8464087

[B9] Huang K. Yang T. Xu J. Yang L. Zhao J. Zhang X. (2019). Prevalence, risk factors, and management of asthma in China: A national cross-sectional study. Lancet 394 (10196), 407–418. 10.1016/S0140-6736(19)31147-X 31230828

[B10] Jordi G. D. David D. P. Carme H. Roberto C. (2020). Study to evaluate satisfaction with the inhalation device used by patients with asthma or chronic obstructive pulmonary disease and the association with adherence and disease control. J. Aerosol Med. Pulm. Drug Deliv. 33 (3), 153–160. 10.1089/jamp.2019.1541 31834826

[B11] Jordi G. Pere R. Carme H. Montserrat T. Meritxell P. Ma J. F. (2016). Knowledge and attitudes of nurses in Spain about inhaled therapy: Results of a national survey. J. Aerosol Med. Pulm. Drug Deliv. 29 (1), 86–93. 10.1089/jamp.2014.1198 26168021

[B12] Jose R. J. Oliver A. N. (2019). The importance of inhaler adherence to prevent COPD exacerbations. Med. Sci. (Basel) 7 (4), 54. 10.3390/medsci7040054 30939829 PMC6524014

[B13] Lin J. T. Wang W. Q. Zhou X. Yin K. S. Liu C. T. Wang C. Z. (2018). Trends of asthma control, disease management and perception in China. Zhonghua Jie He He Hu Xi Za Zhi 41 (3), 191–195. 10.3760/cma.j.issn.1001-0939.2018.03.009 29518847

[B14] López-Campos J. L. Quintana Gallego E. Carrasco Hernández L. (2019). Status of and strategies for improving adherence to COPD treatment. Int. J. Chron. Obstruct Pulmon Dis. 14, 1503–1515. 10.2147/COPD.S170848 31371936 PMC6628097

[B15] Maepa H. M. Wong M. L. Menezes C. N. (2019). Evaluation of the knowledge and correct use of metered-dose inhalers by healthcare professionals and medical students in Gauteng Province. Afr. J. Thorac. Crit. Care Med. 25(3), 111. 10.7196/AJTCCM.2019.v25i3.003 PMC8278855 34286261

[B16] Manjulakshmi P. Kadhiravan T. Manju R. Gitanjali B. (2019). Inadequate inhaler technique, an everlasting problem, is associated with poor disease control - a cross sectional study. Adv. Respir. Med. 87 (4), 217–225. 10.5603/ARM.a2019.0021 31476009

[B17] Mark L. L. Will C. José L. I. A. Claus K. Federico L. Lauri L. (2019). Understanding Dry powder inhalers: Key technical and patient preference attributes. Adv. Ther. 36 (10), 2547–2557. 10.1007/s12325-019-01066-6 31478131 PMC6822825

[B18] Pearce C. J. Fleming L. (2018). Adherence to medication in children and adolescents with asthma: Methods for monitoring and intervention. Expert Rev. Clin. Immunol. 14 (12), 1055–1063. 10.1080/1744666X.2018.1532290 30286679

[B19] Sarah A. B. Vicky K. David B. P. Sinthia B. A. (2021). Identifying patients at risk of poor asthma outcomes associated with making inhaler technique errors. J. Asthma 58 (7), 967–978. 10.1080/02770903.2020.1742353 32162572

[B20] Vinita S. Jin-Gun C. Tracy S. John W. Mary R. (2021). Confidence of nurses with inhaler device education and competency of device use in a specialised respiratory inpatient unit. Chronic Respir. Dis. 18, 14799731211002241. 10.1177/14799731211002241 PMC7983467 33739194

[B21] Wang C. Xu J. Yang L. Xu Y. Zhang X. Bai C. (2018). Prevalence and risk factors of chronic obstructive pulmonary disease in China (the China pulmonary health [CPH] study): A national cross-sectional study. Lancet 391 (1013), 1706–1717. 10.1016/S0140-6736(18)30841-9 29650248

[B22] World Health Organization (2007). Global surveillance, prevention and control of chronic respiratory diseases: A comprehensive approach. Available at: http://www.who.int/gard/publications/GARD_Manual/en/index.html (Accessed March 15, 2022).

[B23] Zhong N. Wang C. Yao W. Chen P. Kang J. Huang S. (2007). Prevalence of chronic obstructive pulmonary disease in China: A large, population-based survey. Am. J. Respir. Crit. Care Med. 176, 753–760. 10.1164/rccm.200612-1749OC 17575095

[B24] Zhu B. Wang Y. Ming J. Chen W. Zhang L. (2018). Disease burden of COPD in China: A systematic review. Int. J. COPD 13, 1353–1364. 10.2147/COPD.S161555 PMC5927339 29731623

