# Supplementary material for: Nurses in China lack knowledge of inhaler devices: A cross-sectional study
Source: Front Pharmacol. 2023 Apr 7;14:1152069. doi: 10.3389/fphar.2023.1152069 (PMC10119394; doi:10.3389/fphar.2023.1152069)
Supplement: Supplementary file 1 [file Table1.docx]

Supplemental table 1 Sociodemographic characteristics and adequate knowledge of inhaler devices of 1831 nurses in this questionnaire

| **Socio-demographic characteristics** | **Adequate Knowledge**  **of inhaler device** | | **Chi square** | **P value** |
| --- | --- | --- | --- | --- |
|  | **Yes n(%)** | **No n(%)** |  |  |
| Recoded age in years |  |  |  |  |
| ＜20 years | 4(20.00) | 16(80.00) | **11.901** | **0.008** |
| >20 years, but＜30 years | 94(12.52) | 657(87.48) |  |  |
| >30 years, but＜40 years | 120(19.14) | 507(80.86) |  |  |
| ≥40 years | 73(16.86) | 360(83.14) |  |  |
| Sex |  |  |  |  |
| Male | 0(0) | 24(100.00) | 3.470 | 0.063 |
| Female | 291(16.10) | 1516(83.90) |  |  |
| Region |  |  |  |  |
| Province-level Municipality | 260(16.25) | 1340(83.75) | 3.255 | 0.354 |
| Provincial Capital | 3(6.52) | 43(93.48) |  |  |
| Prefecture-level city | 7(15.22) | 39(84.78) |  |  |
| County-level city or below | 21(15.11) | 118(84.89) |  |  |
| Hospital level |  |  |  |  |
| Tertiary hospital | 117(13.10) | 776(86.90) | **21.405** | **0.000** |
| Secondary hospital | 10(8.20) | 112(91.80) |  |  |
| Community hospital | 164(20.10) | 652(79.90) |  |  |
| Professional title |  |  |  |  |
| Titles below junior title | 43(13.52) | 275(86.48) | **12.537** | **0.006** |
| Junior title | 126(13.86) | 783(86.14) |  |  |
| Intermediate title | 119(20.17) | 471(79.83) |  |  |
| Senior title | 3(21.43) | 11(78.57) |  |  |
| Education |  |  |  |  |
| Senior school or below | 7(8.98) | 71(91.02) | 3.045 | 0.385 |
| Junior college | 125(16.56) | 630(83.44) |  |  |
| Undergraduate | 158(15.93) | 834(84.07) |  |  |
| Postgraduate | 1(16.67) | 5(83.33) |  |  |
| Working years |  |  |  |  |
| ＜5 years | 57(12.81) | 388(87.19) | **9.125** | **0.028** |
| >5years, but＜10 years | 64(14.19) | 387(85.81) |  |  |
| >10 years, but＜20 years | 102(19.39) | 424(80.61) |  |  |
| ≥20 years | 68(16.62) | 341(83.38) |  |  |
| Major in Respiratory medicine |  |  |  |  |
| Never | 174(12.72) | 1194(87.28) | **91.847** | **0.000** |
| Once | 63(18.21) | 283(81.79) |  |  |
| Current | 54(46.15) | 63(53.85) |  |  |

Supplemental table 2 Socio-demographic characteristics and the technique of inhaler use of 1831 nurses in this questionnaire

| **Socio-demographic characteristics** | **Knew operational key points in using inhaler** | | **Chi square** | **P value** |
| --- | --- | --- | --- | --- |
|  | **Yes n(%)** | **No n(%)** |  |  |
| Recoded age in years |  |  |  |  |
| ＜20 years | 5(25.00) | 15(75.00) | 0.035 | 0.998 |
| >20 years, but＜30 years | 187(24.90) | 564(75.10) |  |  |
| >30 years, but＜40 years | 154(24.56) | 473(75.44) |  |  |
| ≥40 years | 106 (24.48) | 327(75.52) |  |  |
| Sex |  |  |  |  |
| Male | 5(20.83) | 19(79.17) | 0.194 | 0.659 |
| Female | 447(24.74) | 1360(75.26) |  |  |
| Region |  |  |  |  |
| Municipality | 401(25.06) | 1199(74.94) | 5.870 | 0.118 |
| Capital city | 5(10.87) | 41(89.13) |  |  |
| Prefecture-level city | 14(30.43) | 32(69.57) |  |  |
| County-level city or below | 32(23.02) | 107(76.98) |  |  |
| Hospital level |  |  |  |  |
| Tertiary hospital | 207(23.18) | 686(76.82) | 10.514 | 0.005 |
| Secondary hospital | 19(15.57) | 103(84.43) |  |  |
| Community hospital | 226(27.70) | 590(72.30) |  |  |
| Title level |  |  |  |  |
| Titles below junior level | 80(25.16) | 238(74.84) | 2.038 | 0.565 |
| Junior level | 212(23.32) | 697(76.68) |  |  |
| Intermediate title | 156(26.44) | 434(73.56) |  |  |
| Senior title | 4(28.57) | 10(71.43) |  |  |
| Education |  |  |  |  |
| High school or below | 17(21.79) | 61(78.21) | 0.368 | 0.832 |
| Junior college | 187(24.77) | 568(75.23) |  |  |
| Undergraduate and above | 248(24.85) | 750(75.15) |  |  |
| Working years |  |  |  |  |
| ＜5 years | 113(25.39) | 332(74.61) | 0.220 | 0.974 |
| >5years, but＜10 years | 112(24.83) | 339(75.17) |  |  |
| >10 years, but＜20 years | 127(24.14) | 399(75.86) |  |  |
| ≥20 years | 100(24.45) | 309(75.55) |  |  |
| Major in Respirology |  |  |  |  |
| Never | 324(23.68) | 1044(76.32) | 17.995 | 0.000 |
| Once | 80(23.12) | 266(76.88) |  |  |
| Current | 48 (41.02) | 69(58.98) |  |  |
